# *Plasmodium ovale* Malaria Acquired in Central Spain

**DOI:** 10.3201/eid0812.020105

**Published:** 2002-12

**Authors:** Juan Cuadros, Maria José Calvente, Agustin Benito, Juan Arévalo, Maria Angeles Calero, Javier Segura, Jose Miguel Rubio

**Affiliations:** * Hospital Príncipe de Asturias, Alcalá de Henares, Madrid, Spain; † Consejería de Sanidad, Comunidad de Madrid, Madrid, Spain; ‡ Instituto de Salud Carlos III, Madrid, Spain

**Keywords:** Malaria, epidemiology, transmission, prevention, diagnosis, Spain, anopheles, polymerase chain reaction, airport, human

## Abstract

We describe a case of locally acquired *Plasmodium ovale* malaria in Spain. The patient was a Spanish woman who had never traveled out of Spain and had no other risk factors for malaria. Because patients with malaria may never have visited endemic areas, occasional transmission of malaria to European hosts is a diagnostic and clinical challenge.

In the first decades of the 20th century, malaria was a highly endemic disease in Spain. After the civil war (1936–1939), a large epidemic occurred; more than 293,000 cases and 1,278 deaths were reported (1). As a result, public health officials in Spain established strict control measures, and the disease was officially declared eradicated in 1964 (1). Malaria in Spain has been historically transmitted by Anopheles atroparvus and An. labranchiae. However, in recent entomologic surveys conducted in areas that were previously malarious, only An. atroparvus has been found in high densities similar to those observed during the years malaria was endemic (2,3).

 Since the eradication of malaria in Europe, locally acquired malaria on the continent has usually been classified as “airport” or “odyssean” malaria (transmitted by infected mosquitoes transported by airplanes, ships, containers, luggage, buses, and the like) (4,5), and cases of malaria reported in Europe without identifiable risk factors have been classified as “cryptic” malaria (6). These cryptic cases may have occurred through local mosquito-borne transmission. In 1997, a case of malaria was diagnosed in a patient from southern Italy and identified as probable malaria transmitted by an autochthonous mosquito that fed on a gametogenic host; information on this case was shared with public health officials in Europe to reduce the risk of reintroducing malaria into the Mediterranean basin (7).

Because of its close proximity to Africa, Spain is one of the European countries most susceptible to the traffic of sub-Saharan migrant workers and the risk for transmission by mosquitoes migrating from other countries or indigenous anophelines infected by gametogenic hosts has increased substantially. We describe a case of malaria in a European woman who had never traveled out of Spain and had no other risk factors for malaria.

## The Study

 In March 2001, a 75-year-old woman was admitted to the Hospital Príncipe de Asturias in Madrid with a history of intermittent fever for 1 week and no obvious infection. Intravenous treatment with ciprofloxacin was prescribed to treat provisionally diagnosed pyelonephritis. While in hospital, the patient had two episodes of high fever (39°C–40°C) separated by 48-hour intervals with hypoxemia and deterioration of her general condition. On day 7 of fever, the hematologist advised the physician of the presence of rings inside the patient’s erythrocytes (parasitemia rate <1 %). A rapid antigen detection test (HRP2 detection; ICT Diagnostics, Amrad Corporation, Victor, Australia) was done; the test returned negative results for *Plasmodium falciparum* and *P. vivax*. The sample was later identified as *P. ovale* through microscopy and molecular studies at a reference malaria laboratory. Initial treatment with chloroquine followed by primaquine eliminated the infection successfully, and the patient recovered fully without complications.

*P. ovale* was confirmed by semi-nested multiplex polymerase chain reaction (PCR) (8). DNA isolation was carried out with Chelex (9) and the total DNA with the QIAamp DNA Blood Mini Kit (QIAGEN GmbH, Hilden, Germany); the sample was amplified, and a 499-bp fragment compatible with *P. ovale* was determined. PCR was repeated by using primers at two different temperatures, and the presence of *P. ovale* fragments was confirmed. The *P. ovale* fragments, initially embedded in the agarose gel, were then extracted with the help of the column for DNA purification. The rDNA was also amplified by using four specific primers (two forward and two reverse) at two different temperatures to obtain a large quantity of DNA for posterior sequencing. The product of amplification was sequenced in the ABISPRISM 377 XL DNA automatic sequencer (PE Applied Biosystems, Foster City, CA). Afterwards, sequences were sent to the GenBank database; in all the cases, 100% homology for the small subunit of the *P. ovale* rRNA gene was confirmed.

### Epidemiologic Study

 The patient had never traveled outside of Spain nor had any previous contact with people who had lived in or visited a country with endemic malaria. She had not received any packages from malaria-endemic areas. Because of obesity and instability from normotensive hydrocephalus, she had been confined to her home since January 2000, except for two visits to the hospital. She resides in an urban area close to two rivers (<1 km distance) and two international airports (Torrejón de Ardoz [4 km distance] and Barajas [18 km distance]). The city in which the patient lives (Alcalá de Henares) is very close to the Spanish capital, Madrid (30 km), an area with a meso-Mediterranean climate characterized by 400–500 mm of annual rainfall and average temperatures of 6°C in winter and 22°C–24°C in summer. The city’s geographic conditions are semiarid with 3–4 months of dry seasons. Alcalá has 180,060 inhabitants, including 12,711 (7%) foreign residents; 1,121 (0.6 %) of the residents are Africans from malaria-endemic countries located mostly in the western and central regions of Africa.

We investigated the patient’s medical history for other risk factors for malaria. The patient’s uterus, ovaries, gall bladder, and appendix were removed >30 years ago, and she had never received any blood transfusions or blood derivates. Other risk factors such as needle-sharing, malariotherapy, or organ transplants were discarded. The possibility of iatrogenic transmissions during previous hospital admissions (the last visit was 3 weeks before onset of symptoms) was also investigated and ruled out.

In the city in which the patient resides, the incidence of malaria in 1999 and 2000 was 1.2 and 3.7 cases per 100,000 inhabitants, respectively, but no cases were produced by *P. ovale*. In contrast, in the region surrounding Madrid, which includes two international airports, the incidence of imported malaria in 1999 and 2000 was, 2.7 and 3.4 cases per 100,000 inhabitants, respectively, (139 and 171 cases in total); *P. ovale* was the species implicated in 2.1% and 5.3% of these regional cases, respectively. Health care in Spain is free for foreign residents, but they must be inscribed in the local census bureau, and many illegal foreign migrant or temporary workers are likely not covered by any health insurance.

## Conclusions

 This case is the first locally acquired *P. ovale* infection connected with Europe. The infection may have been transmitted by an odyssean vector. The patient lives 4 and 18 km away from international airports, within the radius of other previously reported airport malaria cases (4,12). The case may also have been transmitted by a local mosquito (introduced malaria) (5). In Spain, a possible vector for local infection is *An. atroparvus*, since this species has shown receptivity to *P. vivax* (10) and possibly could be receptive to *P. ovale* as well. The infection could have also been transmitted by an odyssean African vector such as *An. gambiae* (4). Surprisingly, the illness began when cold temperatures started. Thus, the disease could have been a relapse from hepatic hypnozoite or a primoinfection produced by the bite of an inhouse hibernating infected *Anopheles* spp. female (11). Whatever the mechanism, the diagnosis was complicated by the fact that the disease occurred during the winter.

Spain hosts a growing number of migrant workers from west and central Africa traveling through the Gibraltar Strait. Tourism and international flights to and from tropical countries have multiplied in recent years (12). All these factors can account for occasional or epidemic reintroduction of malaria into the country.

Although some studies indicate that indigenous mosquitoes such as *An. labranquiae* and *An. atroparvus* are not susceptible to the afrotropical *P. falciparum* strains (2,10), these species of anophelines are probably fully susceptible to infection by *P. vivax* and *P. ovale* strains imported from Africa. In addition, Asian or American *P. falciparum* strains may also be imported.

 In Europe, malaria transmission can also occur in urban settings given the appropriate conditions. Malaria should be considered in patients with a fever of unknown origin, even if they have never traveled to malaria-endemic areas. Increased attention should be given to persons who work or live close to international airports or in areas with high population of new foreign residents from malarious areas. Hospital laboratories should be ready to detect malaria parasitemia on a 24-hour basis (thick films or antigen detection if microscopy expert is unavailable) on a physician’s request. Likewise, PCR techniques should be used to detect retrospectively low parasitemias and confirm the species diagnosis. We suggest that epidemiologic and clinical attention should be given to travelers and newly arrived foreign residents from malaria-endemic countries to prevent secondary cases (13). Simple, easy access to health care for recently arrived migrant workers should be implemented to assess risk factors and screen for malaria if necessary.
